# New Insights into the Microbial Degradation of *D*-Cyphenothrin in Contaminated Water/Soil Environments

**DOI:** 10.3390/microorganisms8040473

**Published:** 2020-03-26

**Authors:** Yaohua Huang, Ziqiu Lin, Wenping Zhang, Shimei Pang, Pankaj Bhatt, Eldon R. Rene, Alagarasan Jagadeesh Kumar, Shaohua Chen

**Affiliations:** 1 State Key Laboratory for Conservation and Utilization of Subtropical Agro-bioresources, Guangdong Province Key Laboratory of Microbial Signals and Disease Control, Integrative Microbiology Research Centre, South China Agricultural University, Guangzhou 510642, China; 20183138021@stu.scau.edu.cn (Y.H.); 20192047010@stu.scau.edu.cn (Z.L.); 20191047008@stu.scau.edu.cn (W.Z.); 20192047012@stu.scau.edu.cn (S.P.); pankajbhatt.bhatt472@gmail.com (P.B.); 2 Guangdong Laboratory for Lingnan Modern Agriculture, Guangzhou 510642, China; 3 Department of Environmental Engineering and Water Technology, IHE Delft Institute for Water Education, 2601DA Delft, The Netherlands; e.raj@un-ihe.org; 4 School of Chemistry and Chemical Engineering, Jiangsu University, Zhenjiang 212013, China; jaga.jagadeesh1987@gmail.com

**Keywords:** D-cyphenothrin, degradation pathway, kinetics, *Staphylococcus succinus*, bioaugmentation

## Abstract

Persistent use of the insecticide D-cyphenothrin has resulted in heavy environmental contamination and public concern. However, microbial degradation of D-cyphenothrin has never been investigated and the mechanism remains unknown. During this study, for the first time, an efficient D-cyphenothrin-degrading bacterial strain *Staphylococcus succinus* HLJ-10 was identified. Response surface methodology was successfully employed by using Box-Behnken design to optimize the culture conditions. At optimized conditions, over 90% degradation of D-cyphenothrin (50 mg·L^−1^) was achieved in a mineral salt medium within 7 d. Kinetics analysis revealed that its half-life was reduced by 61.2 d, in comparison with the uninoculated control. Eight intermediate metabolites were detected in the biodegradation pathway of D-cyphenothrin including *cis*-D-cyphenothrin, *trans*-D-cyphenothrin, 3-phenoxybenzaldehyde, α-hydroxy-3-phenoxy-benzeneacetonitrile, *trans*-2,2-dimethyl-3-propenyl-cyclopropanol, 2,2-dimethyl-3-propenyl-cyclopropionic acid, *trans*-2,2-dimethyl-3-propenyl-cyclopropionaldehyde, and 1,2-benzenedicarboxylic acid, dipropyl ester. This is the first report about the degradation of D-cyphenothrin through cleavage of carboxylester linkage and diaryl bond. In addition to degradation of D-cyphenothrin, strain HLJ-10 effectively degraded a wide range of synthetic pyrethroids including permethrin, tetramethrin, bifenthrin, allethrin, and chlorempenthrin, which are also widely used insecticides with environmental contamination problems. Bioaugmentation of D-cyphenothrin-contaminated soils with strain HLJ-10 substantially enhanced its degradation and over 72% of D-cyphenothrin was removed from soils within 40 d. These findings unveil the biochemical basis of a highly efficient D-cyphenothrin-degrading bacterial isolate and provide potent agents for eliminating environmental residues of pyrethroids.

## 1. Introduction

D-cyphenothrin [cyano-(3-phenoxyphenyl) methyl 2,2-dimethyl-3-(2-methyl-1-propenyl) cyclopropanecarboxylate] is one of the most popular synthetic pyrethroids (SPs), used for the control a broad-spectrum of insect pests [1]. Lower toxicity and higher insecticidal potential has made D-cyphenothrin highly suitable for indoor control of sanitary pests (cockroaches, mosquitoes, houseflies, fleas, and mites) [2,3,4,5]. However, persistent use of D-cyphenothrin affects non-target organisms and contaminates terrestrial and aquatic environments [6]. Agricultural countries produce large amounts of agro-wastewater that is directly discharged into natural channels, posing serious environmental effects [7]. In the aqueous phase, D-cyphenothrin is separated from water due to non-polar property and adsorbs in sediments [8]. Indigenous microorganisms metabolize these pyrethroids through the hydrolysis of ester bond and oxidation of microsomal cytochrome P450s [9,10]. Despite this, some studies have reported that the concentration of D-cyphenothrin detected frequently was higher than the acceptable daily intake (ADI) [11].

Humans spend most of their lives indoors and are exposed to various indoor pollutants including D-cyphenothrin [12,13]. Long-term exposure to pyrethroids can affect human health. Stiller-Winkler et al. have demonstrated decreased concentrations of humoral and cellular immune function parameters with the increased exposure of pesticide applicators to SPs [14]. Previous studies have indicated that prolonged D-cyphenothrin exposure may cause toxicity to the human reproductive system [15]. A survey conducted by Le Grand et al. detected pyrethroid metabolite 3-phenoxybenzoic acid (3-PBA) in the urine samples of all 39 French volunteers who were not exposed to SPs [16]. The concentration of metabolites was noted to be higher in the urine than blood [17]. These results reveal potential hazards of SPs, including D-cyphenothrin, to human health and ecosystems.

The persistent use of D-cyphenothrin and its environmental residues has raised serious concerns. Therefore, there is an urgent need to develop effective strategies to solve D-cyphenothrin related problems. Bioremediation is a cost-effective, eco-friendly, and highly efficient approach to eliminate organic contaminants and pyrethroid residues [18,19,20]. In recent years, several pyrethroid-degrading bacteria have been reported to utilize SPs as a sole carbon source for their growth. These bacteria include *Pseudomonas fulva* P31, *Streptomyces aureus* HP-S-01, *Bacillus subtilis* BSF01, and *Rhodopseudomonas* sp. PSB07-6 [21,22,23,24,25,26,27]. Researchers have also studied pyrethroid-degrading fungi such as *Aspergillus* sp. CBMAI 1829, *Penicillium raistrickii* CBMAI 931, and *Cladosporium* sp. HU [28,29,30]. Microorganisms-based SPs degradation causes ester cleavage to form 3-PBA which is further metabolized [31]. Carboxylesterases-based hydrolysis of SPs and other ester-containing compounds has already been documented [32,33].

To date, microbial degradation of D-cyphenothrin has never been investigated. In this study, D-cyphenothrin biodegradation experiments were performed with the following objectives: (Ⅰ) to isolate and characterize the D-cyphenothrin-degrading strain from activated sludge, (Ⅱ) to optimize D-cyphenothrin biodegradation conditions using response surface methodology, (Ⅲ) to estimate the degradation kinetics of D-cyphenothrin and other SPs, (Ⅳ) to identify the intermediate metabolites formed during D-cyphenothrin degradation, and (Ⅴ) to investigate the bioremediation of D-cyphenothrin in sterilized and unsterilized soil environments. 

## 2. Materials and Methods

### 2.1. Chemicals and Medium

D-cyphenothrin (98% purity) was obtained from Wuhan Yuancheng Pharm Co., Ltd., China. Technical-grade tetramethrin (99%), permethrin (97%), bifenthrin (96%), allethrin (93%), and chlorempenthrin (94%) were purchased from Sigma-Aldrich, USA. Chromatographic grade acetone and acetonitrile was obtained from Fisher Scientific, USA. All other chemicals and solvents used in this study were of analytical grade. Stock solutions were prepared by dissolving SPs in chromatographic grade acetone at a concentration of 10 g·L^−1^ and stored in dark bottles at 4 °C.

Luria-Bertani medium (LB) was composed of 10 g tryptone; 5 g yeast extract; and 10 g NaCl dissolved in 1-L distilled water. Mineral salt medium (MSM) (g·L^−1^) containing (NH_4_)_2_SO_4_, 2; Na_2_HPO_4_·12H_2_O, 1.5; KH_2_PO_4_, 1.5; MgSO_4_·7H_2_O, 0.2; CaCl_2_·2H_2_O, 0.01; and FeSO_4_·7H_2_O, 0.001 was used for biodegradation assays whereas solid medium was additionally added with 1.5% agar. The pH of both culture media was adjusted to 7.0 and autoclaved at 121 °C for 20 min.

### 2.2. Isolation and Identification of D-Cyphenothrin-Degrading Bacterial Isolates

Pesticide activated sludge was collected from a farmland in Harbin, Heilongjiang Province, China. Five g of soil sample was added to a 250 mL Erlenmeyer flask containing 50 mL of sterilized MSM medium. Bacterial culture was enriched by adding D-cyphenothrin stock in the flask to make a final concentration of 50 mg∙L^−1^. Flask was incubated at 30 °C and 200 rpm in a rotary shaker. After 7 d, 5 mL culture was transferred into a new flask containing 50 mL fresh MSM medium at a concentration of 100 mg·L^−1^. The same method was continuously repeated to prepare enrichment culture concentrations of 200, 400, and 800 mg·L^−1^, respectively. Final enrichment culture (800 mg·L^−1^) was serially diluted and spread on MSM plates (1.5% agar) containing D-cyphenothrin (50 mg·L^−1^). Plates were incubated at 30 °C for 3 d to isolate individual colonies. Individual colonies were selected and purified by re-streaking three times, and preserved with 15% glycerol at −80 °C. D-cyphenothrin degradation potential of the isolate was determined by high-performance liquid chromatography (HPLC) (Waters, Milford, MA, USA).

Bacterial strain HLJ-10 was identified and characterized by morphology, physio-biochemical characteristics, and genetic analysis of 16S rDNA gene sequence. After two days of incubation at 30 °C and 200 rpm, morphological characteristic of strain HLJ-10 (size, color, surface, edge, and texture) were studied under light microscope (Olympus, Tokyo, Japan) and scanning electron microscope (XL-30 ESEM, Philips Optoelectronics Co., Ltd., Holland) [34]. Physio-biochemical assays were conducted with reference to previous literature [35]. The 16S rRNA gene was PCR-amplified with universal set of primer pairs (forward primer: 5′-TGACGAGTGGCGGACGGGTG-3′ and reverse primer: 5′-CCATGGTGTGACGGGCGGTGTG-3′) purchased from Genewiz, NJ, USA. The resulting sequence of PCR product (1136 bp) was compared in Basic Local Alignment Search Tool (BLAST) of National Center for Biotechnology Information (NCBI). Multiple alignment of 16S rRNA were performed in DNAMAN (version 6.0, LynnonBiosoft, San Ramon, CA, USA) and phylogenetic tree was constructed in MEGA 7.0 (Pennsylvania State University, University Park, PA, USA) by following the neighbor-joining method according to Tamura et al. [36].

### 2.3. Optimization of Conditions for D-Cyphenothrin Degradation 

Stored culture (−80 °C) of strain HLJ-10 was thawed and activated by streaking on LB agar plates. Two days later, single colonies were inoculated into LB liquid medium for 24 h. After adjusting the OD (optical density) to 0.8, 1 mL of bacterial broth was taken in a 2 mL centrifuge tube and centrifuged (4000× *g*, 4 min) to collect the cells. The cells were washed twice with sterile normal saline and re-suspended. Re-suspended broth was transferred to 50 mL MSM medium containing D-cyphenothrin (50 mg·L^−1^) and incubated in the dark at 30 °C and 200 rpm. The experiment was set up in triplicate and uninoculated flasks served as controls. The growth OD of strain HLJ-10 was monitored with UV/visible spectrophotometer (Shimadzu UV-2410, Shimadzu, Japan) at 600 nm and residual amount of D-cyphenothrin was determined by HPLC.

Response surface methodology (RSM) designed by Box–Behnken was used to optimize the crucial factors and interactive influences of strain HLJ-10 degradation activity. According to the preliminary one-factor-at-a-time experiment results, three main factors (temperature, pH, and inoculum size) were selected as independent variables to achieve optimal conditions for D-cyphenothrin degradation. Range and center values of the three independent variables are shown in Table 1. Degradation of 50 mg·L^−1^ D-cyphenothrin in MSM after 7 d was considered as the dependent variable. Box–Behnken design with three-variables consisting of 17 experimental runs in triplicate at the midpoint was generated by Design Expert version 12.0 (Stat-Ease, MN, USA). Randomized block design was followed and experimental data were subjected to regression analysis. The data were analyzed by response surface regression program and fitted to the following quadratic polynomial equation:

*Y_i_= b_0_ +∑ b_i_X_i_ + ∑ b_ij_X_i_X_j_ + ∑ b_ii_X_i_^2^* (1)

where *Y_i_* is the predicted response, *X_i_* and *X_j_* are variables, *b_0_* is the constant, *b_i_* is the linear coefficient, *b_ij_* is the interaction coefficient, and *b_ii_* is the quadratic coefficient.

To confirm the effect of strain HLJ-10 on D-cyphenothrin degradation, a first-order kinetic model was followed to describe the biodegradation process:

(2) $$C_{t}=C_{0}\times e^{-\mathrm{kt}}$$

where *C*_0_ is the initial concentration of D-cyphenothrin (mg∙L^−1^), *C*_t_ is the content of D-cyphenothrin at time *t*, *k* is the degradation constant (day^−1^), and *t* is the degradation time (days).

Equation (3) was used to calculate the theoretical half-life (t_1/2_) of D-cyphenothrin: $t_{1/2}=\frac{\ln(2)}{k}$

*t*_1/2_ = ln2/*k* (3)

where ln (2) is the natural logarithm of 2 and *k* is the degradation constant.

### 2.4. Substrate Range of Strain HLJ-10 and Concentration Range of D-Cyphenothrin

The degradation potential of strain HLJ-10 was studied against various SPs (permethrin, bifenthrin, tetramethrin, allethrin, and chlorempenthrin) and different concentrations of D-cyphenothrin. Various SPs were added to the 50 mL of MSM medium at a final concentration of 50 mg∙L^−1^, respectively. D-cyphenothrin was added to the 50 mL MSM medium to make a final concentration of 25, 50, 100, 200, 400, and 800 mg∙L^−1^, respectively. The experiments were performed in triplicate with uninoculated flasks as controls. After 7 d of shaking in dark at 30 °C and 200 rpm, the samples were extracted and analyzed by HPLC.

### 2.5. Identification of Intermediate Metabolites of D-Cyphenothrin

To study the metabolic pathway of strain HLJ-10 during D-cyphenothrin degradation, samples were collected from MSM on day 1, 3, 5, and 7, respectively. Metabolites were detected by gas chromatography-mass spectrometry (GC/MS) (Agilent 6890N/5975, Santa Clara, CA, USA) and results were matched with standard compounds in the National Institute of Standards and Technology (NIST) library database.

### 2.6. Biodegradation of D-Cyphenothrin in Soils

To analyze the effect of strain HLJ-10 in soil, an experiment with sterile and nonsterile soil was planned. Soil samples for this experiment were collected from the top 5–20 cm layer of a field located in South China Agricultural University, Guangdong Province, China where D-cyphenothrin had never been applied. Physicochemical parameters for the soil were characterized as (g/kg of dry weight): organic matter, 10.5; total N, 0.5; total P, 0.4; total K, 18.2; and pH, 6.9. Soil consisted of sand, 65.0%; silt, 28.0%; and clay, 7.0%. Soil samples were naturally air dried indoors and sieved (2 mm) for bioremediation studies [18].

Soil was autoclaved at 121 °C for 1 h to completely remove indigenous microbial communities. Then, 250 g sterile and nonsterile soil samples were placed in a 500 mL Erlenmeyer flask, respectively. D-cyphenothrin solution was added to a final concentration of 50 mg∙kg^−1^ in each flask. Soil moisture content was adjusted to 40%–50% with sterile deionized water. Approximately 6.0 × 10^8^ CFU∙mL^−1^ bacterial cell suspension was inoculated to sterile and nonsterile soils, respectively, whereas uninoculated samples served as the control. After complete mixing, soil remediation experiments were carried out in the dark at 30 °C. Then, 10 g soil samples were collected on 5th, 10th, 15th, 25th, and 40th day for extraction.

### 2.7. Extraction method of D-Cyphenothrin

To extract D-cyphenothrin from MSM medium, a 50 mL centrifuge tube was used instead of the traditional separatory funnel. Then, 10 mL of the collected sample was added to a 50 mL centrifuge tube and 10 mL acetone was added. Tubes were vortexed for 10 s followed by ultrasonication for 20 min. Then, 15 mL ethyl acetate was added to the tube and vortexed for 1 min. Tubes were kept at room temperature until the aqueous and organic phases were clearly layered. Upper organic phase was transferred to a new 50 mL centrifuge tube, 15 mL ethyl acetate was re-added, and the same procedure was repeated. Organic phases were combined in the new tube and original tubes were discarded. About 10 g of anhydrous sodium sulfate was added to the tube containing organic phase, vortexed for 30 s, and kept for 20 min. Subsequently, the organic phase was transferred to a 100 mL flat-bottom flask and dried by vacuum evaporation. D-cyphenothrin residues and metabolites were recovered by chromatographic acetonitrile. Recovered samples were further collected by using 1 mL injector and 0.22 μm filter membrane and stored in brown bottles at 4 °C before HPLC detection.

### 2.8. Analytical Methods

D-cyphenothrin was quantified with Waters 2690 HPLC system equipped with a Phenomenex C_18_ reverse phase column (250 nm × 4.60 mm, 5 μm) and UV detector. Mobile phase was composed of acetonitrile and deionized water (75:25) at a flow rate of 1.0 mL·min^−1^. Injection volume and detection wavelength were 10 μL and 253 nm, respectively. Retention times of D-cyphenothrin, permethrin, bifenthrin, tetramethrin, allethrin, and chlorempenthrin remained as 8.97, 14.04, 20.99, 6.78, 7.22, and 10.78 min, respectively.

D-cyphenothrin metabolites were identified in Agilent 6890N/5975 GC/MS system equipped with auto-sampler, an on-column, split/splitless capillary injection system, and HP-5MS capillary column (30.0 m × 250 μm × 0.25 μm) with array detector. Helium was used as carrier gas at a flow rate of 1.5 mL·min^−1^. Analytical mode was scanned from 30–500 nm. The column temperature was first held at 90 °C for 2 min, raised at 6 °C·min^−1^ to 150 °C for 1 min, 10 °C·min^−1^ to 180 °C for 4 min, and finally 20 °C·min^−1^ to keep at 260 °C for 10 min. Temperatures corresponding to transfer line and ion source were 280 and 230 °C, respectively. The column outlet was directly inserted into electron ionization source block at 70 eV. The injection volume was 1.0 μL with splitless sampling at 250 °C [37].

## 3. Results and Discussion

### 3.1. Isolation and Characterization of Strain HLJ-10

Activated sludge collected from Harbin, Heilongjiang Province, had a large number of isolates with various characteristics. Isolates were obtained in MSM agar plates containing 50 mg∙L^−1^ D-cyphenothrin. One of the isolates utilized D-cyphenothrin as a carbon and energy source to grow and was designated as HLJ-10. Gram staining characterized this isolate as Gram-positive strain. Scanning electron microscope (SEM) revealed that strain HLJ-10 was nearly ball-shaped having a diameter of approximately 0.7–1 μm. Strain HLJ-10 colonies appeared light yellow, smooth, moist, opaque, and with irregular edges, on LB plates. Detailed appearance and scanning electron micrograph of the strain HLJ-10 are shown in Figure S1. The optimum degradation temperature and pH were found to be about 32 °C and 8.0 respectively, and could grow with NaCl (up to 15%) in LB broth. Strain HLJ-10 exhibited significant degradation potential in the pH range of 5–11 but failed to grow at pH 3. Physiological and biochemical tests showed positive results in Gram-staining, starch hydrolysis, gelatin liquefaction, and urease tests, whereas negative results were noted in esculin hydrolysis, sorbierite, and raffinose (Table S1). Turbid growth was observed in LB broth medium after 24 h of incubation in shaker at 30 °C and 200 rpm. 

**Figure 1:**
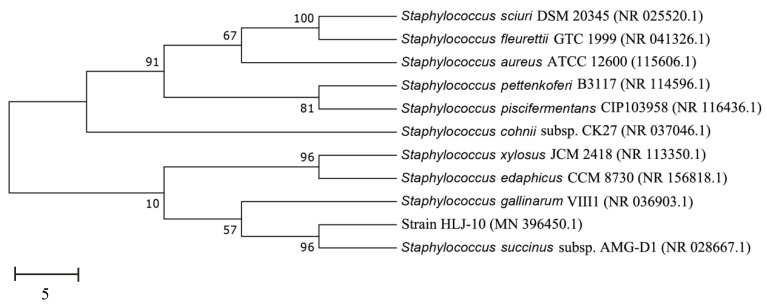
Phylogenetic tree based on 16S rDNA sequences of *Staphylococcus succinus* HLJ-10 and representative *Staphylococcus succinus*. Numbers in parentheses represent sequence accession number in GenBank. Numbers at nodes indicate bootstrap values. Bar represents sequence divergence.

Phylogenetic analysis of 16 S rDNA gene sequence indicated that strain HLJ-10 belongs to the genus *Staphylococcus* and shares high similarity with *S. succinus* subsp. AMG-D1 (GenBank accession number NR028667) (Figure 1). Partial 16S rDNA gene sequence of strain HLJ-10 was submitted to the GenBank under the accession number MN396450. Based on the 16S rRNA analysis and morphology, the strain HLJ-10 was identified as *Staphylococcus succinus*. *S. succinus* is a widespread bacterium in various natural habitats and possesses catabolic capabilities. Several potent microbial genera have been reported for SPs biodegradation including *Micrococcus*, *Ochrobactrum*, *Raoultella*, *Klebsiella*, *Brevibacterium*, *Candida, Bacillus*, and *Pseudomonas* [19,38,39,40,41]. However, degradation of xenobiotic compounds by genus *Staphylococcus* has not received the attention it deserves and this study for the first time presented SPs degradation by *S. succinus*.

### 3.2. Growth of Isolate and Kinetic Analysis of Degradation Process

**Figure 2:**
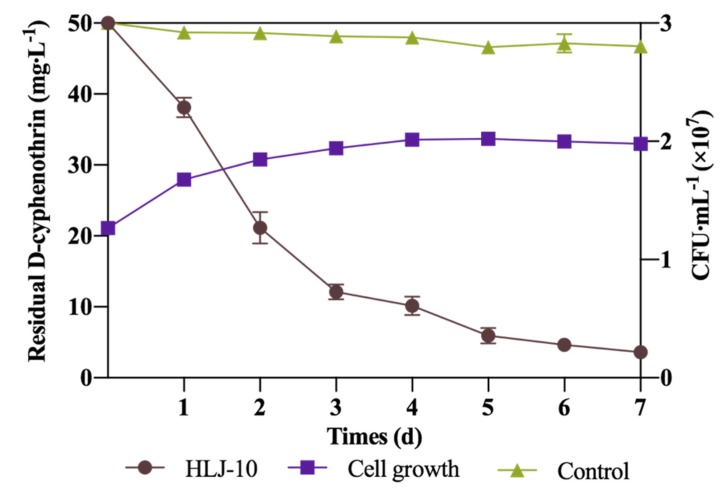
Utilization of D-cyphenothrin during growth of *Staphylococcus succinus* HLJ-10. Error bars indicate standard deviation of three replicates.

Degradation kinetics of D-cyphenothrin and growth of strain HLJ-10 were simultaneously investigated in MSM medium with 50 mg∙L^−1^ D-cyphenothrin. As shown in Figure 2, there was no lag phase during the growth of strain HLJ-10, indicating that it effectively utilized D-cyphenothrin as a growth substance by degrading 75.8% in 72 h. D-cyphenothrin degradation was correlated with bacterial cell density and biodegradation of D-cyphenothrin rapidly increased during exponential phase (72 h), whereas it slowed down in stationary phase. Approximately 92.8% of D-cyphentorhrin was degraded on the 7th day in the dark. HPLC analysis of D-cyphenothrin degradation by strain HLJ-10 over time is shown in Figure S2. At the same time, no significant change in D-cyphenothrin concentration was observed in the control flasks without bacterial inoculum.

Kinetic parameters of D-cyphenothrin degradation by strain HLJ-10 are shown in Table 2, which reveal that the degradation process of D-cyphenothrin followed a first-order kinetic model (Equation (2)). Degradation constant (*k*) of strain HLJ-10 and control were noted as 0.3896 and 0.011, respectively. Theoretical *t*_1/2_ value of the treatments with strain HLJ-10 and controls were calculated by Equation (3). Determination coefficients (*R*^2^) of strain HLJ-10 and control were found as 0.9659 and 0.983, respectively, indicating that degradation data were well fitted with first-order kinetic model. The *t*_1/2_ of the D-cyphenothrin degradation by strain HLJ-10 was determined as 1.8 d, which is significantly shorter than 63.0 d of control. These results further illustrated efficiency of strain HLJ-10 in D-cyphenothrin degradation.

To date, there are only few reports about microbial degradation of D-cyphenothrin. Suzuki et al. recently reported the metabolic behavior of D-cyphenothrin in aqueous environments but D-cyphenothrin-degrading microorganisms were not specified [31]. It is noteworthy that most of pyrethroid-degrading microbes isolated could not grow in the absence of extra carbon sources [42]. Strain HLJ-10 directly utilized D-cyphenothrin as the sole carbon and energy source for its growth that exhibited its promising potential for the bioremediation of pyrethroid-contaminated environments.

### 3.3. Optimization of D-Cyphenothrin Degradation Conditions

Experimental design variables corresponding to D-cyphenothrin degradation are presented in Table 3. Data were analyzed by response surface regression in Design Expert 12.0. Experimental values of D-cyphenothrin residues were fitted to the following quadratic polynomial model equations (Equation (4):

*Y*_1_ = 94.46 − 1.6*X*_1_ − 2.1*X*_2_ − 0.85*X*_3_ + 0.2*X*_1_*X*_2_ + 4.1*X*_1_*X*_3_ − 1.15*X*_2_*X*_3_ − 7.56*X*_1_^2^ − 10.46*X*_2_^2^ − 1.15*X*_3_^2^ (4)

where *Y*_1_ is the predicted D-cyphenothrin degradation (%); *X*_1_, *X*_2_, and *X*_3_ are coded values of temperature, pH, and inoculum size, respectively.

Table 4 presents analysis of variance (ANOVA) to fit a quadratic polynomial model. Significant *p* value (0.0277) of the model term indicates that the equation reliably predicted D-cyphenothrin degradation. A low coefficient of variation (CV = 5.41%) also suggested that the model is accurate and reliable. Results of regression analysis revealed that linear and square terms of temperature (*X*_1_) and pH (*X*_2_) had significant (*p* < 0.05) effect on D-cyphenothrin degradation by strain HLJ-10, whereas the linear term of inoculum size (*X*_3_) and interaction terms were insignificant (*p* > 0.05). These results were similar to the previous report of Chen et al. [37], who degraded cypermethrin by a co-culture of *Bacillus cereus* ZH-3 and *Streptomyces aureus* HP-S-01.

**Figure 3:**
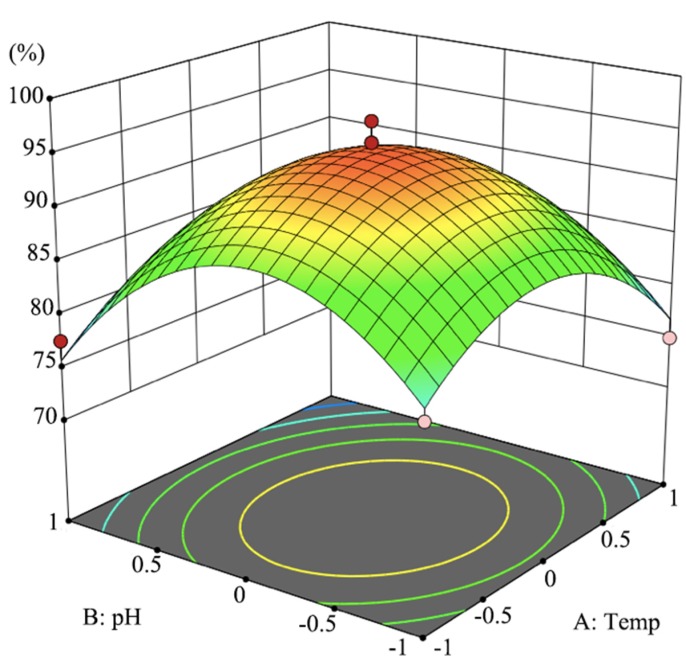
Response surface plot showing the effects of pH and temperature (°C) on D-cyphenothrin degradation by *Staphylococcus succinus* HLJ-10 while fixing the zero of inoculum size at a coded level.

The inoculum size was fixed at OD 0.8 (6.98 × 10^8^ CFU/mL) and a three-dimensional response surface was drawn to visually present the effect of temperature and pH on D-cyphenothrin degradation by strain HLJ-10. As shown in Figure 3, the plot of D-cyphenothrin degradation had a maximum theoretical value of 90.7% at a stationary point, whereas optimal levels of three variables *X*_1_, *X*_2_, and *X*_3_ were noted as 2.3, 1.9, and −0.06 at coded levels. The optimum temperature, pH, and inoculum size at the unencoded levels were found as 31.6 °C, 7.9, and 6.05 × 10^8^ CFU∙mL^−1^, respectively. Strain HLJ-10 was capable of rapidly degrading D-cyphenothrin without a lag phase over a wide range of temperature (25–35 °C) and pH (5–9). This is a very important feature of an organism for bioremediation of variable environments [30]. Results demonstrated that degradation tends to occur under alkaline conditions which is consistent with SPs characteristic of easy hydrolyzation under alkaline conditions [43]. On the other side, acidity attenuated the activity of *S. succinus* HLJ-10, which resulted in decreased D-cyphenothrin degradation.

### 3.4. Degradation Products and Degradation Pathway of D-Cyphenothrin

The degradation pathway of D-cyphenothrin by strain HLJ-10 was performed through GC/MS. Two significant peaks were detected at retention times of 27.975 and 28.050 min, showing a characteristic mass fragment [M^+^] at *m/z* 375 with same major fragment ions at *m/z* 123 and were confirmed as two isomers of the same compound. The compound was designated as compound A having isomers 1 and 2. These two isomers of compound A were further characterized as *cis*-D-cyphenothrin and *trans*-D-cyphenothrin according to the fragment retention time (RT) and the similarity of molecular ions to corresponding authentic compounds in the National Institute of Standards and Technology (NIST, USA) library database. As the concentration of D-cyphenothrin was decreased, six new compounds B, C, D, E, F, and G were detected. Chemical structures, RTs, and characteristic ions of the mass spectra (*m/z*) are listed in Table 5. RTs of compounds B, C, D, E, F, and G were noted as 23.16, 23.14, 23.39, 16.81, 16.76, and 26.63 min, respectively. Based on the similarity of fragment RTs and molecular ions to those of corresponding authentic compounds in the NIST library database, compounds B, C, D, E, F, and G were identified as *trans*-2,2-dimethyl-3-propenyl-cyclopropanol, 2,2-dimethyl-3-propenyl-cyclopropionic acid, *trans*-2,2-dimethyl-3-propenyl-cyclopropionaldehyde, α-hydroxy-3-phenoxy-benzeneacetonitrile, 3-phenoxybenzaldehyde, and 1,2-benzenedicarboxylic acid and dipropyl ester, respectively (Figure S3). Mass spectrum of the matched D-cyphenothrin metabolites in the NIST library database are shown in Figure S4. 

**Figure 4:**
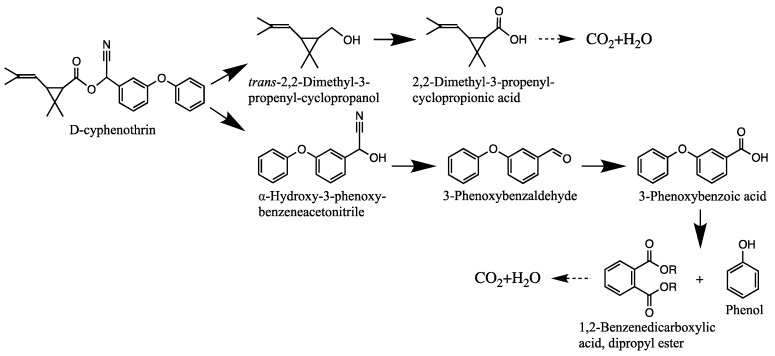
Proposed D-cyphenothrin degradation pathway in *Staphylococcus succinus* HLJ-10.

Based on the GC/MS results, the microbial degradation pathway of D-cyphenothrin was first time proposed (Figure 4). D-cyphenothrin was first hydrolyzed by the cleavage of carboxylester linkage to yield α-hydroxy-3-phenoxy-benzeneacetonitrile and *trans*-2,2-dimethyl-3-propenyl-cyclopropanol. Intermediate product α-hydroxy-3-phenoxy-benzeneacetonitrile was unstable in the environment and spontaneously converted to 3-phenoxybenzaldehyde. Subsequently, 3-phenoxybenzaldehyde was further degraded through diaryl cleavage to form 1,2-benzenedicarboxylic acid and dipropyl ester. Meanwhile, *trans*-2,2-dimethyl-3-propenyl-cyclopropanol was oxidized to form *trans*-2,2-dimethyl-3-propenyl-cyclopropionaldehyde, which was transient and further oxidized to 2,2-dimethyl-3-propenyl-cyclopropionic acid. At the end of experiment, all these metabolites faded away without any non-cleavable metabolites. In the non-inoculated controls with the same quantity of D-cyphenothrin, only D-cyphenothrin was detected after 7 d.

Among these compounds, α-hydroxy-3-phenoxy-benzeneacetonitrile and 3-phenoxybenzaldehyde have been frequently detected in SPs biodegradation [18,35]. These intermediates were transient with very low concentrations and easily transformed into smaller molecular compounds through oxidation and hydrolysis. Microbial degradation of pyrethroids mainly involves the hydrolysis of ester bonds and oxidation reaction [23]. Carboxylesterase enzyme is produced by the microorganism that specifically cleaves a carboxylate bond [44,45]. This cleavage transforms parent pesticide into the smaller molecular weight compound containing a carboxylic acid or alcohol that further oxidizes and dehydrogenates to yield less toxic or non-toxic compounds [46,47]. It is worth noting that the D-cyphenothrin degradation products are non-toxic to the environment.

### 3.5. Degradation of Various SPs and Different Concentrations of D-Cyphenothrin

**Figure 5:**
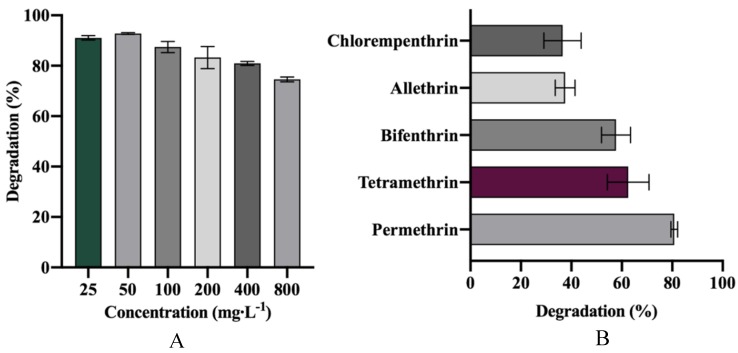
(**A**) Degradation of different initial concentrations of D-cyphenothrin by *Staphylococcus succinus* HLJ-10; (**B**) Degradation of various pyrethroids by *Staphylococcus succinus* HLJ-10.

In order to study the degradation potential of strain HLJ-10 under different environments, degradation experiments were carried out with different concentrations of D-cyphenothrin (Figure 5A) and 50 mg∙L^−1^ of various SPs (Figure 5B). Initial concentrations of D-cyphenothrin were prepared as 25, 50, 100, 200, 400, and 800 mg∙L^−1^, respectively. The highest degradation (92.8%) was noted at 50 mg∙L^−1^. Unexpectedly, at 25 mg∙L^−1^, D-cyphenothrin was not completely degraded and maximum degradation reached 91.8%. Strain HLJ-10 rapidly degraded D-cyphenothrin at concentrations up to 800 mg∙L^−1^ and utilized it as a sole carbon and energy source to grow without any lag period. At a D-cyphenothrin concentration of 800 mg∙L^−1^, maximum degradation reached 74.6% after 7 d. Previous studies revealed that the degradation activity of pyrethroid-degrading bacterial strains was substantially inhibited at high SPs concentrations [48,49]. This particular strain was found highly effective in degrading D-cyphenothrin up to the concentration as high as 800 mg·L^−1^, indicating that strain HLJ-10 may be suitable for the bioremediation of various contaminated environments.

Generally, multiple SPs are used in rotation instead of repeating a single pyrethroid for a longer time. Therefore, residues of various SPs are found in terrestrial and aquatic environments [50,51]. Different SPs including tetramethrin, permethrin, bifenthrin, allethrin, and chlorempenthrin were separately added to the MSM medium at a final concentration of 50 mg∙L^−1^. Degradation of permethrin was found to be the highest (80.8%), followed by tetramethrin (62.5%). The lowest degradation occurred in allethrin and chlorempenthrin as 37.5% and 36.5%, respectively. Usually, the degraders only transform a single xenobiotic compound due to certain structural requirements [40]. Strain HLJ-10 was able to degrade a wide range of SPs, suggesting that the isolate possesses promising potential and advantages in removing pyrethroid residues from various environments.

### 3.6. Biodegradation of D-Cyphenothrin in Soils

**Figure 6:**
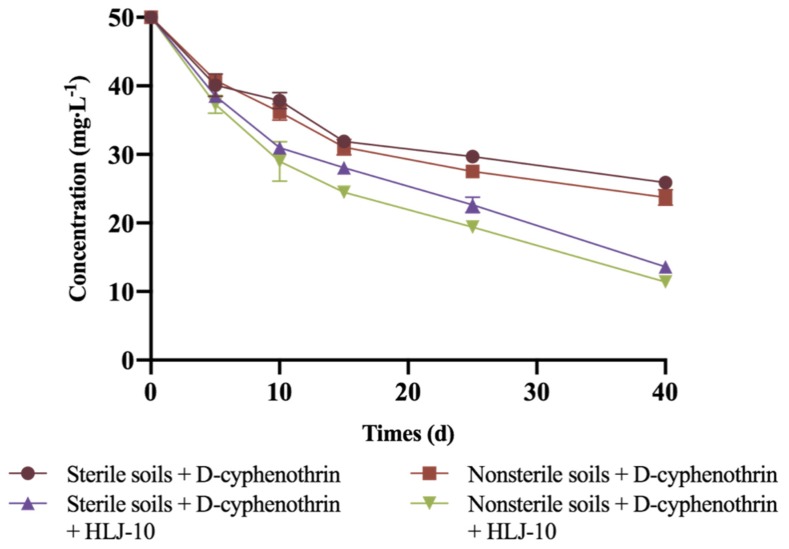
Degradation kinetics of D-cyphenothrin in different soils.

Soil remediation experiments were carried out under sterile and nonsterile conditions, whereas non-inoculated treatment served as the control (Figure 6). After bioremediation with strain HLJ-10, approximately 77.2% and 72.8% of the initially added D-cyphenothrin was eliminated in nonsterile and sterile soils, respectively, within 40 d. Degradation parameters were characterized as *k* values of 0.0323 and 0.0283 day^−1^ and *t*_1/2_ values of 21.5 and 24.5 d, respectively. Specific kinetic parameters are shown in Table 6. In addition, no significant lag phase was observed during the experiment. In the controls with indigenous microbiota, 52.5% of D-cyphenothrin was removed, which is slightly higher than sterilized control (48.2%) and *t*_1/2_ values were noted as 46.2 and 55.5 d, respectively, indicating that indigenous microbiota play a role in the bioaugmentation of pyrethroid-contaminated sites. Similar results were observed by Zhan et al. [35]. Nonsterile soil contains natural microbial communities, which led to a higher degradation rate.

Several pyrethroid-degrading microbes were reported and effectively removed pyrethroids under optimum conditions in liquid media, but few were subjected to soil remediation [33,40,42,52,53]. The degrading microbes isolated from the environment usually fail to degrade xenobiotics when used for bioremediation of contaminated soils; thus, additional treatments are needed to accelerate biodegradation [23,43,54]. In this work, bioaugmentation of D-cyphenothrin-contaminated soils with strain HLJ-10 substantially enhanced the disappearance rate of D-cyphenothrin, and its *t*_1/2_ was reduced by 31.0 and 24.7 d in sterile and nonsterile soils, respectively, in comparison with soils without the strain HLJ-10. Unlike most bacterial isolates, strain HLJ-10 has an exceptional ability to adapt and thrive in different ecological niches, which makes it a potent strain for various applications. 

However, it is worth noting that the strain HLJ-10 exhibited a significant reduction in the ability to remediate soil compared with the liquid medium experiment. Microorganisms in the soil are more likely to be affected by factors such as pH, soil water content, organic matter content, soil porosity, heavy metals, and other organic contaminants than by liquid media [46,55,56,57]. To improve the ability of strain HLJ-10 to remediate in soil, these issues should be fully considered.

## 4. Conclusions

In this study, we identified a novel bacterial isolate *S. succinus* HLJ-10 having superb D-cyphenothrin degradation activity. Strain HLJ-10 utilized D-cyphenothrin as the sole carbon source for its growth over a wide range of temperature (25–35 °C) and pH (5–9), indicating that the strain has excellent working ability in adverse environments. This study proves that the bacterium harbors the metabolic pathway for complete detoxification and metabolism of D-cyphenothrin. This is the first report of the D-cyphenothrin degradation pathway that is of vital importance in the D-cyphenothrin biogeocycle. Further, this particular strain exhibited great advantages in bioremediation of D-cyphenothrin-contaminated soils because of its adaptability in different environments. Furthermore, this strain was capable of degrading a wide range of SPs, suggesting that strain HLJ-10 is a potent and effective candidate for the bioremediation of pyrethroid-contaminated terrestrial and aquatic environments. However, further studies, such as its interaction with environment, degradation enzymes, and genes that encode for key enzymes, are still needed before the application of this strain in the field-scale bioremediation. 

## Figures and Tables

**Table 1:** The code and levels of three independent variables used in the central composite rotatable design.

Independent Variables	Code	Code Levels of Variables		
		−1	0	1
Temperature (°C)	*X*_1_	25	30	35
pH	*X*_2_	5	7	9
Inoculum size (OD)	*X*_3_	0.3	0.8	1.3

**Table 2:** Kinetic parameters of D-cyphenothrin degradation with *Staphylococcus succinus* HLJ-10 in mineral salt medium (MSM).

Treatments	Regression Equation	*k*	*R* ^2^	*t*_1/2_
Control	*C_t_* = 49.8*e*^−0.011*t*^	0.011	0.9830	63.0
HLJ-10	*C_t_* = 50.2*e*^−0.3896*t*^	0.3896	0.9659	1.8

Note: *k* refers to degradation constant (day^−1^); *t*_1/2_ refers to half-time (days); *R*^2^ refers to determination coefficient.

**Table 3:** Box–Behnken experimental design matrix and response of dependent variable for D-cyphenothrin degradation.

Run	*X*_1_	*X*_2_	*X*_3_	*Y*_1_
1	0	0	0	92.8
2	0	0	0	94.9
3	1	1	0	74.1
4	0	0	0	94.9
5	1	0	1	91.6
6	1	0	−1	77.4
7	−1	0	1	85.9
8	0	−1	1	82.9
9	0	0	0	96.9
10	−1	0	−1	88.1
11	0	1	1	73.4
12	0	1	−1	85.1
13	−1	−1	0	79.2
14	1	−1	0	74.9
15	0	−1	−1	90.0
16	−1	1	0	77.6
17	0	0	0	92.8

Note: *X*_1_ = Temperature; *X*_2_ = pH; *X*_3_ = Inoculum size; *Y*_1_ = Degradation (%).

**Table 4:** Analysis of variance (ANOVA) of fitted quadratic polynomial model for D-cyphenothrin degradation.

Source	DF	SS	MS	*F*-Value	*p*-Value^*^
Model	9	891.53	99.06	4.64	0.0277
*X*_1_	1	20.48	20.48	0.9595	0.3599
*X*_2_	1	35.28	35.28	1.65	0.2395
*X*_3_	1	5.78	5.78	0.2708	0.6188
*X*_1_ *X*_2_	1	0.1600	0.1600	0.0075	0.9334
*X*_1_ *X*_3_	1	67.24	67.24	3.15	0.1192
*X*_2_ *X*_3_	1	5.29	5.29	0.2478	0.6339
*X*_1_ *X*_1_	1	240.33	240.33	11.26	0.0122
*X*_2_ *X*_2_	1	460.24	460.24	21.56	0.0024
*X*_3_ *X*_3_	1	5.62	5.62	0.2632	0.6237
Residual	7	149.41	21.34		
Lack of Fit	3	137.56	45.85	15.48	0.0115
Pure Error	4	11.85	2.96		
Cor Total	16	1040.94			

Note: *X*_1_ = Temperature; *X*_2_ = pH; *X*_3_ = Inoculum size. DF refers to degrees of freedom; SS refers to sum of sequences; MS refers to mean square. ^*^p level < 0.05 indicates that model terms are significant.

**Table 5:** Chromatographic properties of D-cyphenothrin metabolites during degradation with *Staphylococcus succinus* HLJ-10.

Code	RT (min)	*m/z*	Compound Structure	Name
A_1_	27.975	375	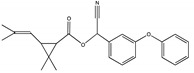	(*cis*) D-cyphenothrin
A_2_	28.050	375	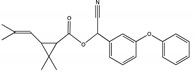	(*trans*) D-cyphenothrin
B	23.160	156	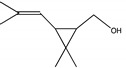	*trans*-2,2-Dimethyl-3-propenyl-cyclopropanol
C	23.144	172	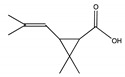	2,2-Dimethyl-3-propenyl-cyclopropionic acid
D	23.399	154	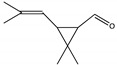	*trans*-2,2-Dimethyl-3-propenyl-cyclopropionaldehyde
E	16.814	227	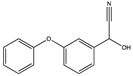	α-Hydroxy-3-phenoxy-benzeneacetonitrile
F	16.765	198	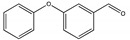	3-Phenoxybenzaldehyde
G	26.634	232	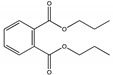	1,2-Benzenedicarboxylic acid, dipropyl ester

**Table 6:** Kinetic parameters of D-cyphenothrin degradation with *Staphylococcus succinus* HLJ-10 in sterile and nonsterile soils.

Treatments	Regression Equation	*k*	*R* ^2^	*t*_1/2_
Sterile soils + D-cyphenothrin	*C_t_* = 50.3*e*^−0.011*t*^	0.0124	0.9302	55.9
Nonsterile soils + D-cyphenothrin	*C_t_* = 51.8*e*^−0.3896*t*^	0.015	0.942	46.2
Sterile soils + D-cyphenothrin + HLJ-10	*C_t_* = 51.2*e*^−0.3896*t*^	0.0283	0.9878	24.5
Nonsterile soils + D-cyphenothrin + HLJ-10	*C_t_* = 52.1*e*^−0.3896*t*^	0.0323	0.9899	21.5

*k* refers to degradation constant (day^−1^); *t*_1/2_ refers to half-time (days); *R*^2^ refers to determination coefficient. Note: like other footnote.

## Supplementary Materials

The following are available online at https://www.mdpi.com/2076-2607/8/4/473/s1. Figure S1: External morphology and scanning electron micrograph of strain HLJ-10. Pictures a (6420×) and b (21880×) are scanning electron micrographs. Picturesc and d are front and back sides of strain HLJ-10 grown on LB agar plates. Figure S2: HPLC analysis of D-cyphenothrin degradation by strain HLJ-10 over time. Figure S3: Mass spectrum of GC-MS identified for D-cyphenothrin intermediates. A1 and A2 were isomers of D-cyphenothrin, and B to G were *trans*-2,2-dimethyl-3-propenyl-cyclopropanol, 2,2-dimethyl-3-propenyl-cyclopropionic acid, *trans*-2,2- dimethyl-3-propenyl-cyclopropionaldehyde, α-hydroxy-3-phenoxy-benzeneacetonitrile, 3-phenoxybenzaldehyde, and 1,2-benzenedicarboxylic acid, dipropyl ester, respectively. Figure S4: The mass spectra of D-cyphenothrin metabolites reported in the National Institute of Standards and Technology (NIST, USA) library database. (a) *trans*-2,2-dimethyl-3-propenyl-cyclopropanol; (b) 2,2-dimethyl-3-propenyl-cyclopropionic acid; (c) *trans*-2,2-dimethyl-3-propenyl-cyclopropionaldehyde; (d) α-hydroxy-3-phenoxy-benzeneacetonitrile; (e) 3-phenoxybenzaldehyde; (f) 1,2-benzenedicarboxylic acid, dipropyl ester. Table S1: Physiological and biochemical characteristics of strain HLJ-10.
