# Supplementary material for: New Insights into the Microbial Degradation of D-Cyphenothrin in Contaminated Water/Soil Environments
Source: Microorganisms. 2020 Mar 26;8(4):473. doi: 10.3390/microorganisms8040473 (PMC7232362; doi:10.3390/microorganisms8040473)
Supplement: Supplementary file 1 [file microorganisms-08-00473-s001.pdf]

Supplementary Information

New insights into the microbial degradation of D-cyphenothrin in contaminated water/soil environments

Yaohua Huang[1,2], Ziqiu Lin[1,2], Wenping Zhang[1,2], Shimei Pang[1,2], Pankaj Bhatt[1,2], Eldon R. Rene[3], Alagarasan Jagadeesh Kumar[4], and Shaohua Chen[1,2*]

[1]*State Key Laboratory for Conservation and Utilization of Subtropical Agro-bioresources, Guangdong Province Key Laboratory of Microbial Signals and Disease Control, Integrative Microbiology Research Centre, South China Agricultural University, Guangzhou 510642, China*;

[2]*Guangdong Laboratory for Lingnan Modern Agriculture, Guangzhou 510642, China*;

[3]*Department of Environmental Engineering and Water Technology, IHE Delft Institute for Water Education, 2601DA Delft, The Netherlands*;

[4]*School of Chemistry and Chemical Engineering, Jiangsu University, Zhenjiang 212013, China*

[*]Corresponding author

Shaohua Chen

South China Agricultural University, Guangzhou 510642, China

Tel: +86-20-8528 8229

Fax: +86-20-8528 0292

E-mail: shchen@scau.edu.cn

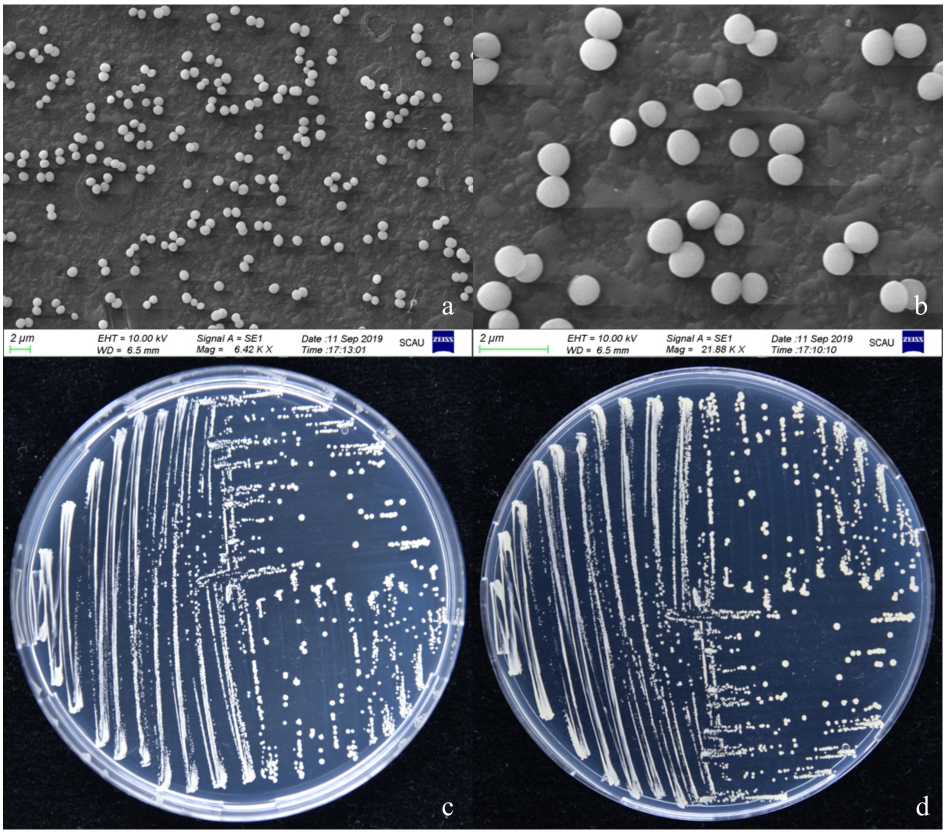

Figure S1. External morphology and scanning electron micrograph of strain HLJ-10. Pictures a (6420×) and b (21880×) are scanning electron micrographs. Picturesc and d are front and back sides of strain HLJ-10 grown on LB agar plates.

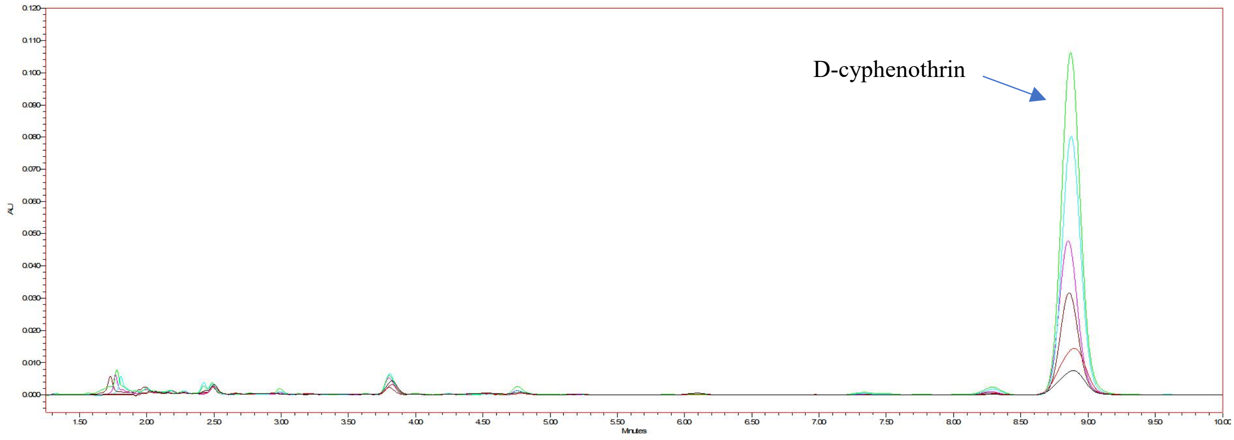

Figure S2. HPLC analysis of D-cyphenothrin degradation by strain HLJ-10 over time

A1
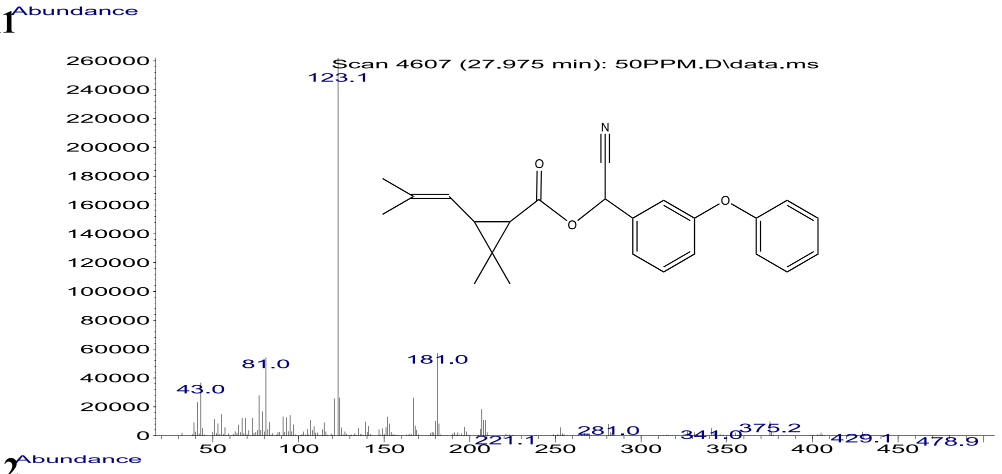
Abundance
Scan 4607 (27.975 min): 50PPM.D\data.ms
123.1
81.0
181.0
43.0
221.1
281.0
341.0
375.2
429.1
478.9
N
O
O
O

A2
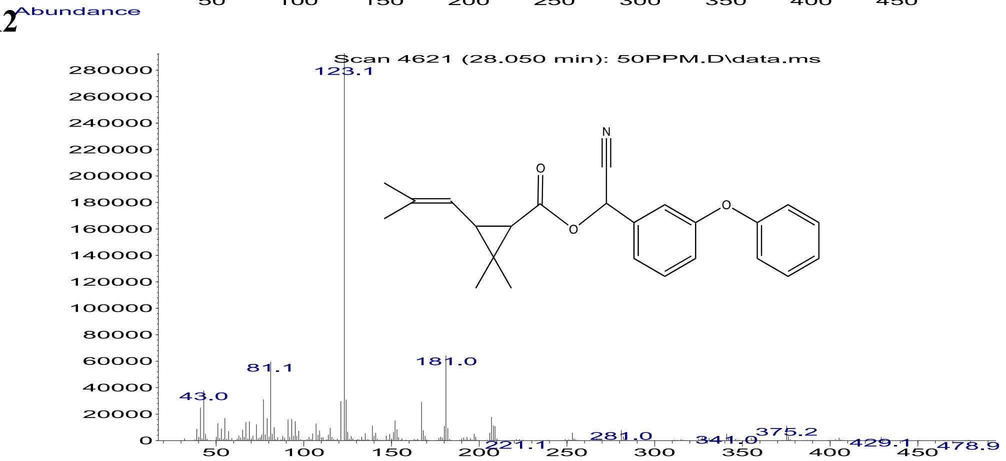
Abundance
Scan 4621 (28.050 min): 50PPM.D\data.ms
123.1
81.1
181.0
43.0
221.1
281.0
341.0
375.2
429.1
478.9
N
O
O
O

B
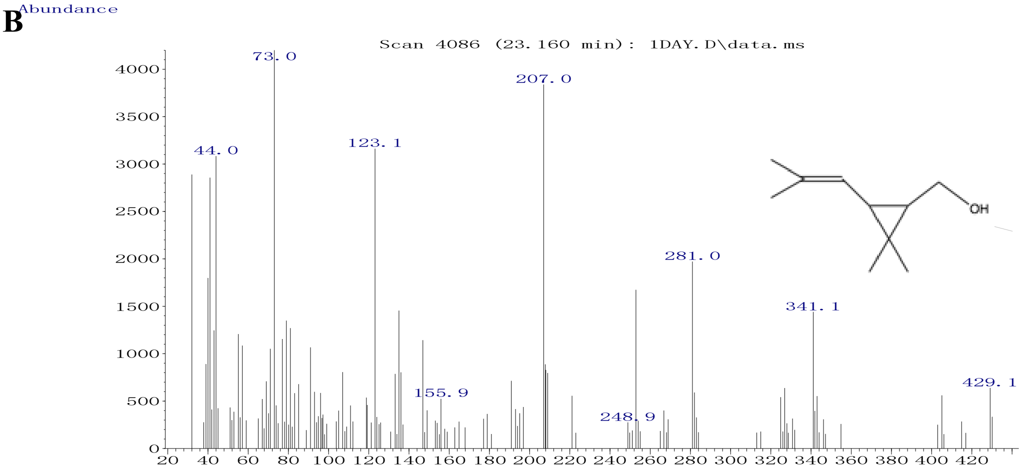
Abundance
Scan 4086 (23.160 min): 1DAY.D\data.ms
73.0
207.0
44.0
123.1
281.0
341.1
155.9
248.9
429.1
OH

C
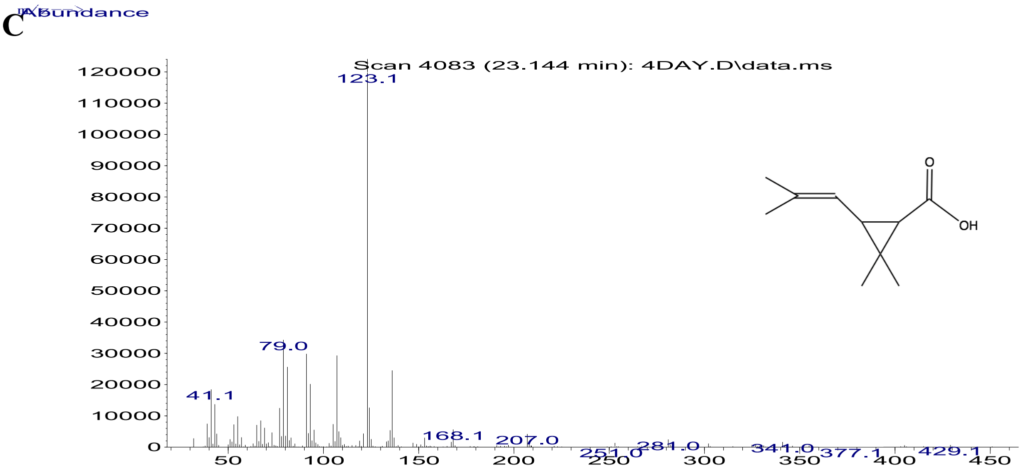
Abundance
Scan 4083 (23.144 min): 4DAY.D\data.ms
123.1
79.0
41.1
168.1
207.0
251.0
281.0
341.0
377.1
429.1
O
OH

D

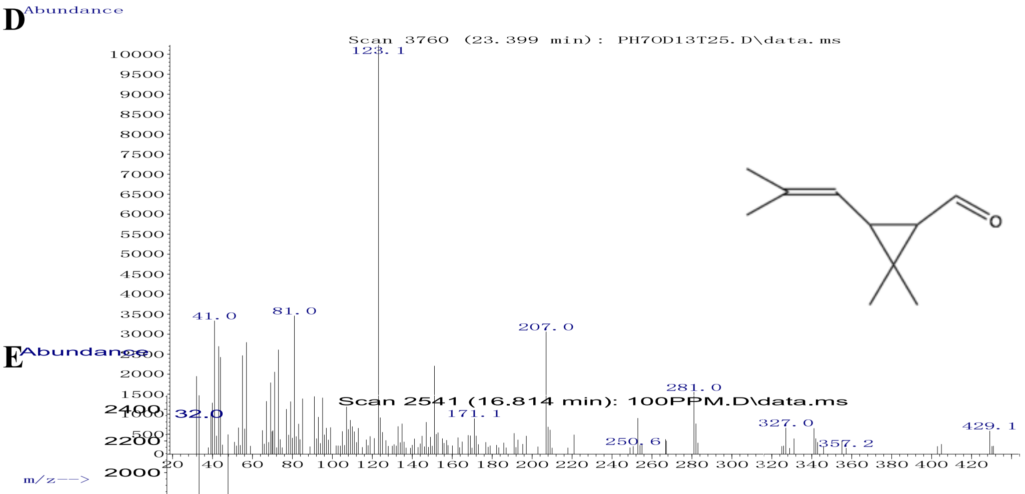
Abundance
Scan 3760 (23.399 min): PH7OD13T25.D\data.ms
123.1
41.0
81.0
207.0
281.0
171.1
250.6
327.0
357.2
429.1
m/z-->

E

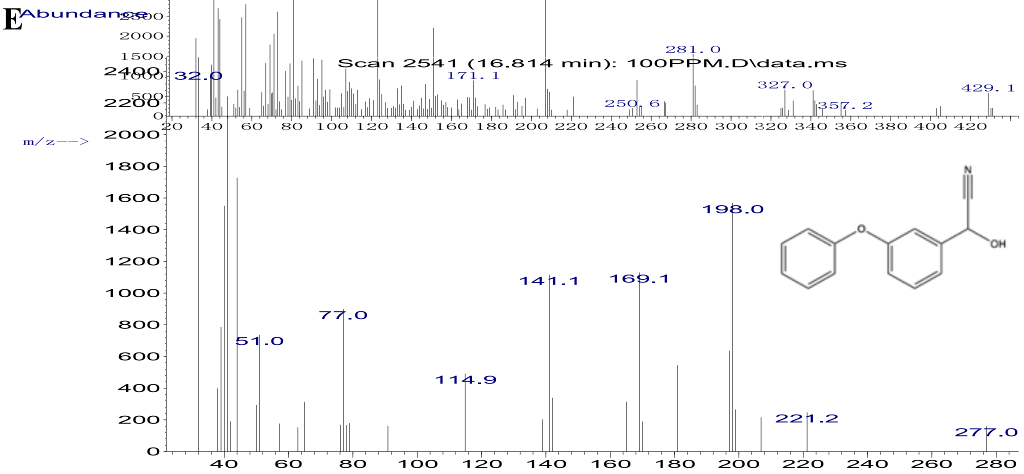
Abundance
Scan 2541 (16.814 min): 100PPM.D\data.ms
32.0
198.0
141.1
169.1
77.0
51.0
114.9
221.2
277.0
N
O
OH

F

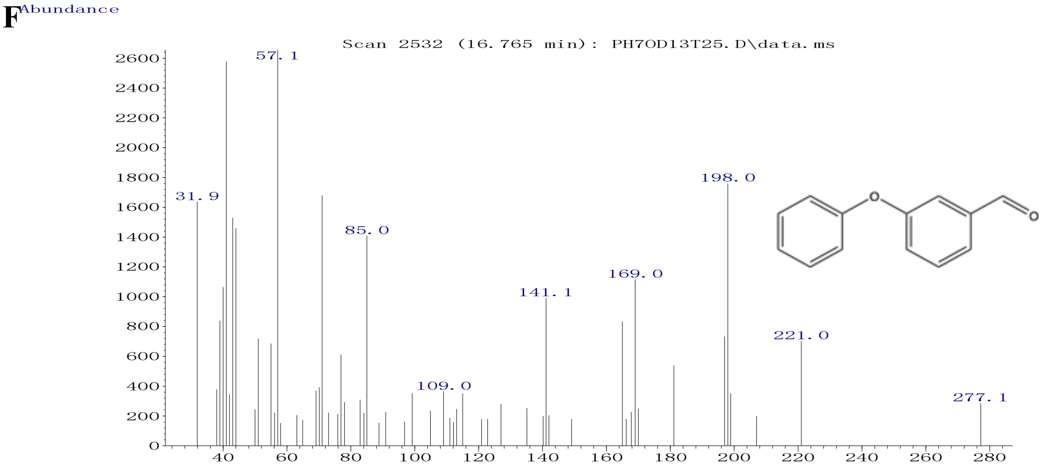
Abundance
Scan 2532 (16.765 min): PH7OD13T25.D\data.ms
57.1
31.9
198.0
85.0
169.0
141.1
221.0
109.0
277.1
O
O

G

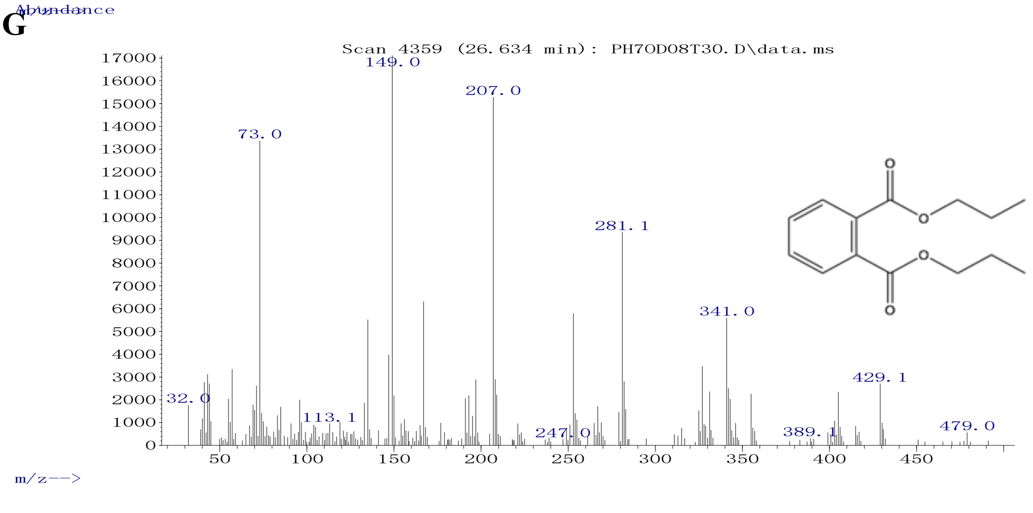
Abundance
Scan 4359 (26.634 min): PH7OD08T30.D\data.ms
149.0
207.0
73.0
281.1
341.0
429.1
32.0
113.1
247.0
389.1
479.0
m/z-->

Figure S3. Mass spectrum of GC-MS identified for D-cyphenothrin intermediates. A1 and A2 were isomers of D-cyphenothrin, and B to G were *trans*-2,2-dimethyl-3-propenyl-cyclopropanol, 2,2-dimethyl-3-propenyl-cyclopropionic acid, *trans*-2,2-dimethyl-3-propenyl-cyclopropionaldehyde, α-hydroxy-3-phenoxy-benzeneacetonitrile, 3-phenoxybenzaldehyde and 1,2-benzenedicarboxylic acid, dipropyl ester, respectively.

a

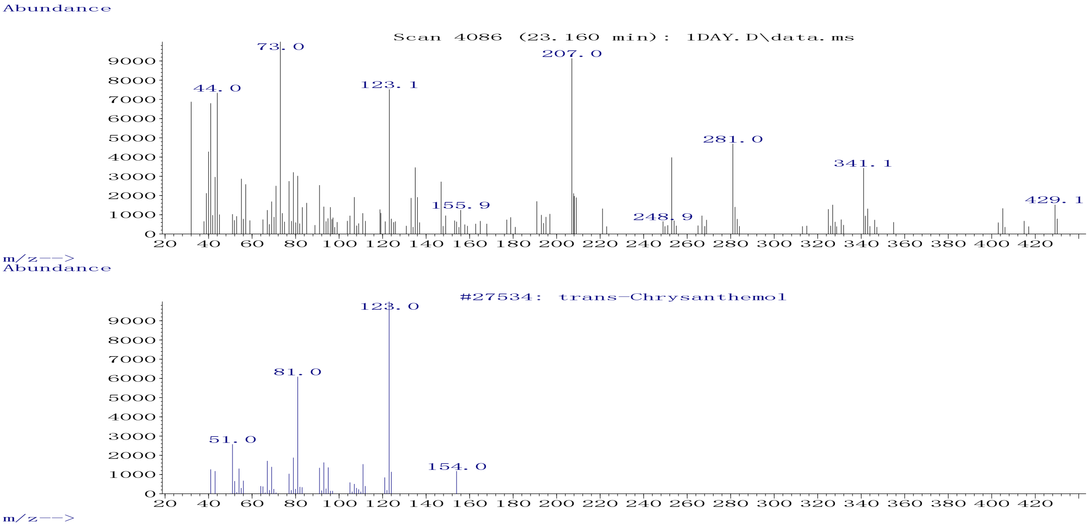
Abundance
Scan 4086 (23.160 min): 1DAY.D\data.ms
73.0
44.0
123.1
207.0
281.0
341.1
155.9
248.9
429.1
m/z-->
Abundance
#27534: trans-Chrysanthemol
123.0
81.0
51.0
154.0
m/z-->

b

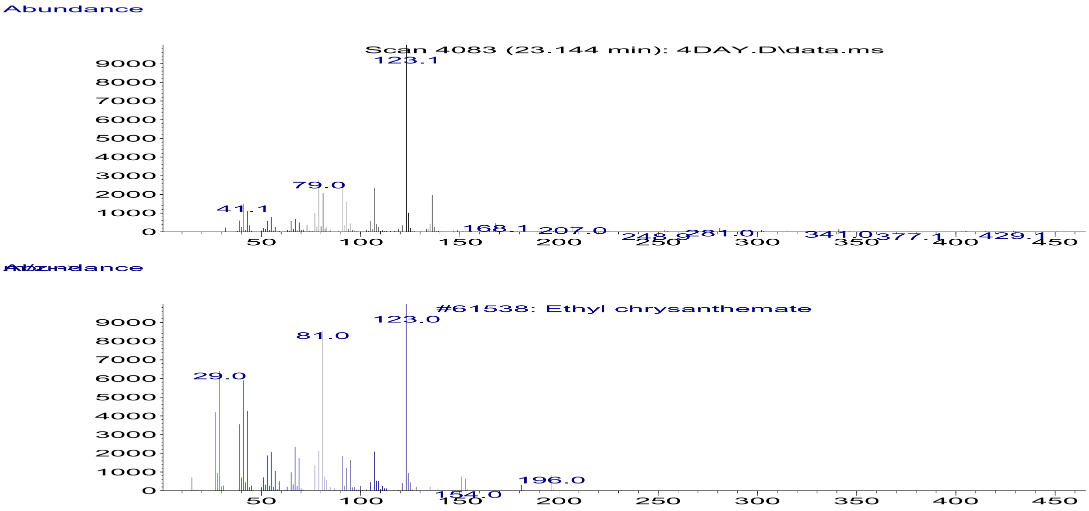
Abundance
Scan 4083 (23.144 min): 4DAY.D\data.ms
123.1
79.0
41.1
168.1
207.0
248.9
281.0
341.0
377.1
429.1
#61538: Ethyl chrysanthemate
123.0
81.0
29.0
196.0
154.0

c

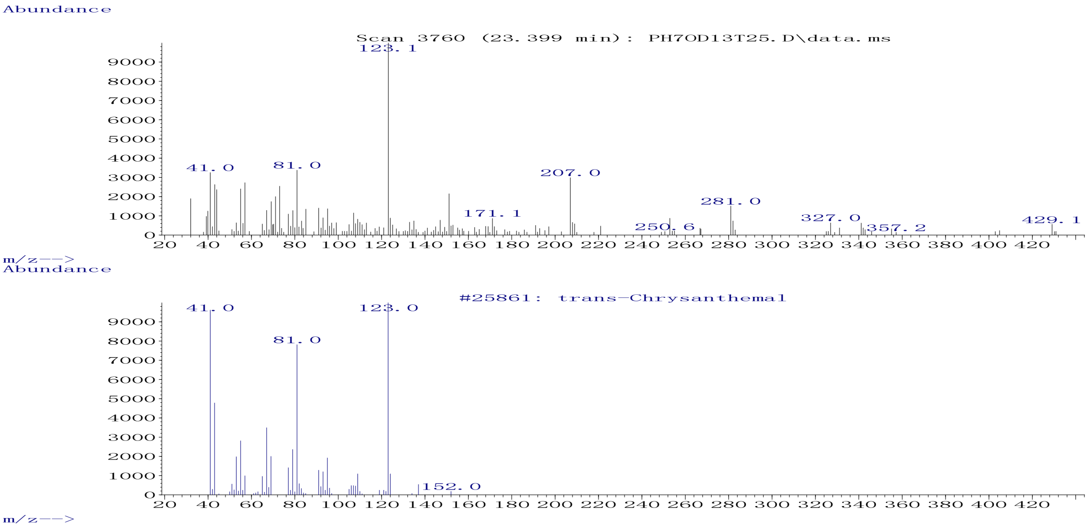
Abundance
Scan 3760 (23.399 min): PH7OD13T25.D\data.ms
123.1
41.0
81.0
207.0
281.0
171.1
327.0
250.6
357.2
429.1
m/z-->
Abundance
#25861: trans-Chrysanthemal
41.0
123.0
81.0
152.0
m/z-->

d

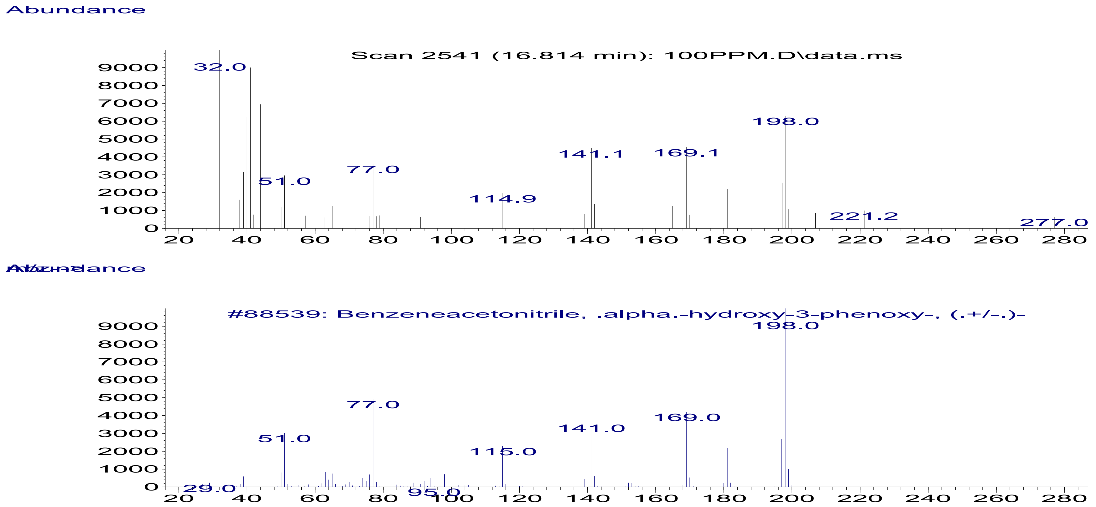
Abundance
Scan 2541 (16.814 min): 100PPM.D\data.ms
#88539: Benzeneacetonitrile, .alpha.-hydroxy-3-phenoxy-, (.+/-.)-

e

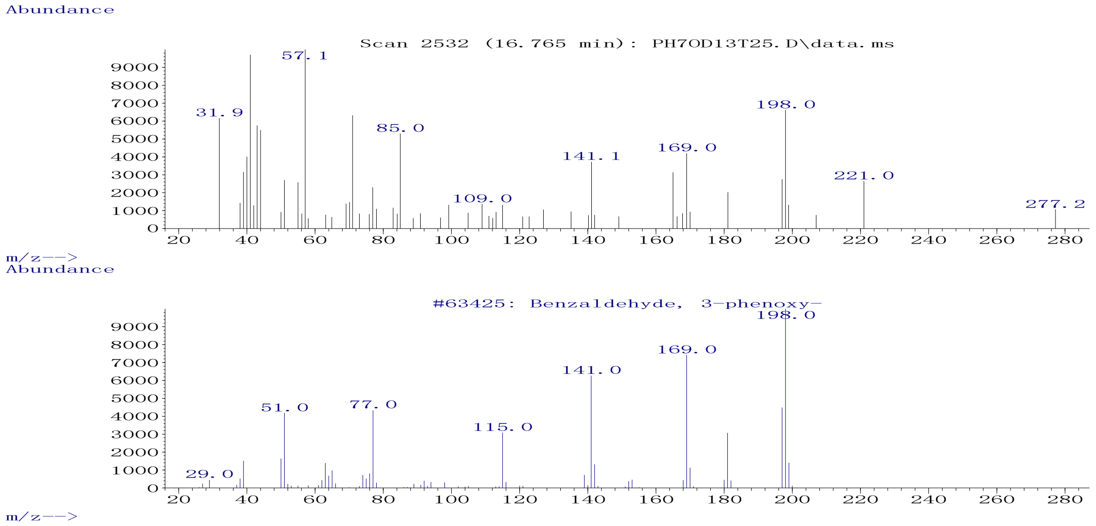
Abundance
Scan 2532 (16.765 min): PH7OD13T25.D\data.ms
m/z-->
#63425: Benzaldehyde, 3-phenoxy-

f

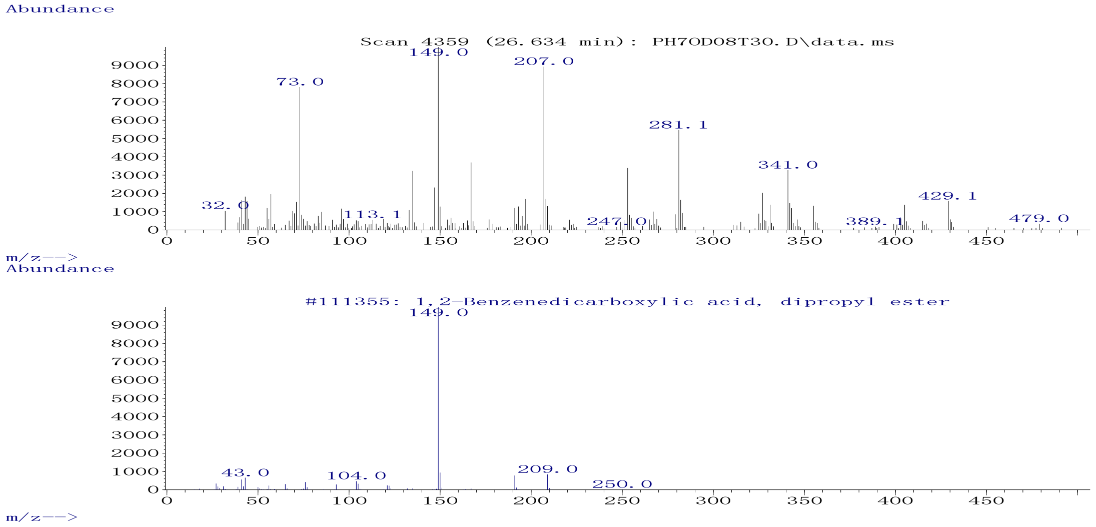
Abundance
Scan 4359 (26.634 min): PH7OD08T30.D\data.ms
m/z-->
#111355: 1,2-Benzenedicarboxylic acid, dipropyl ester

Figure S4. The mass spectra of D-cyphenothrin metabolites reported in the National Institute of Standards and Technology (NIST, USA) library database. (a) *trans*-2,2-dimethyl-3-propenyl-cyclopropanol; (b) 2,2-dimethyl-3-propenyl-cyclopropionic acid; (c) *trans*-2,2-dimethyl-3-propenyl-cyclopropionaldehyde; (d) α-hydroxy-3-phenoxy-benzeneacetonitrile; (e) 3-phenoxybenzaldehyde; (f) 1,2-benzenedicarboxylic acid, dipropyl ester.

Table S1

Physiological and biochemical characteristics of strain HLJ-10

Test	Results	Test	Results
Gram-staining	+	pH 3	−
Sucrose	+	pH 5	+
Glucose	+	pH 7	+
Maltose	+	pH 9	+
Mannitol	+	pH 11	+
Fructose	+	2% NaCl	+
Sorbierite	−	5% NaCl	+
Maltose	+	8% NaCl	+
Trehalose	+	10% NaCl	+
Raffinose	−	15% NaCl	+
Starch hydrolysis	+	8 °C	−
Gelatin liquefaction	+	20 °C	+
Urease	+	30 °C	+
Nitrate reduction	+	40 °C	+
Esculin hydrolysis	−	50 °C	−

Note: "+": tested positive for growth; "−": tested negative for growth.
